# An Account of a Ruptured Uterus

**Published:** 1800

**Authors:** John Sims

**Affiliations:** Physician in London.


XV. *An Account of a ruptured Uterus. By* John Sims, *M. D. Physician in London.*

A Well formed woman, mother of several children, seven months gone with child, after taking a very long walk, under great agitation of mind, was, upon her return home, seized with an uterine hemorrhage, which continued some

ſome days, and then gradually abated, and did not afterwards return ; but the woman continued very weak and ailing for two months, when according to her reckoning ſhe had completed the full period of geſtation. On the 11th of May (1792) ſhe was taken with labour pains, and ſent for her midwife, who gave her expectations of a ſpeedy delivery. But the pains going off, ſhe left her in the evening, with aſſurances that the child preſented right, and that every thing was in a ſafe way. The next morning finding herſelf very ill, but without labour pains, the patient ſent to an experienced practitioner in the neighbourhood, who attended, and, upon examination, found the mouth of the womb not ſufficiently dilated to admit his finger ; he could feel no membranes diſtended with water, nor any part of a child, either through the mouth of the womb, or through the parietes of the womb itſelf. Her face was bloated, her legs and thighs œdematous, and her belly very large. From theſe circumſtances he very rationally ſuſpected that ſhe was not with child, and directed his attention to the hydropic ſymptoms. But as ſhe grew daily worſe, I was deſired to ſee her

 on on the 16th of May, when I found her unable to lie down in bed, complaining of violent pains in her fide; her refpiration was fhort and frequent; her pulfe extremely rapid, with fome hardnefs in the ftroke; a fetid black difcharge flowed from the vagina; her legs and thighs were much fwollen, and pitted upon preffure; the mouth of the womb was relaxed, and a little open at the firft entrance, juft as it is frequently found, when unimpregnated, in women who have had feveral children; no part of a child could be felt through the parietes of the womb, nor could any enlargement of this organ be perceived. I was immediately convinced, that if the midwife had given a true account of the cafe at the time fhe was firft called, a rupture of the uterus had taken place, and the child had efcaped into the cavity of the abdomen. With this idea I examined the ftate of the belly externally, which was very large, and hard to the feel; the tumour circumfcribed as in pregnancy, but nothing like the extremities of a child could be felt through the integuments; and when afterwards the woman was able to lie down, and turn in her bed, I could not find, upon examining in different pofitions, that the tumour fell to the depending fide. The patient, herfelf, complained complained of a ſenſe of greater weight on the one ſide than on the other, but this did not change to the other ſide upon her turning in bed.

She had made no water for more than twenty-four hours before I ſaw her. I ordered her urine to be drawn off with the catheter, which operation was neceſſarily repeated from time to time for about a week after. On account of the urgency of the difficulty of breathing, and the pain in her ſide, eight or nine ounces of blood were directed to be immediately taken from the arm, and a bliſter was applied to the ſide affected; ſhe took an anodyne bolus with rhubarb every night, and a cordial draught every ſix hours.

Upon the 18th, I found her breathing much relieved, the pain in her ſide gone, her pulſe conſiderably more calm, though it ſtill beat 120 ſtrokes in a minute. The diſcharge from the vagina was much increaſed, and moſt intolerably fetid. She was ordered to take a decoction of bark with aromatic confection, and a nouriſhing generous diet; but from a total loſs of appetite, and conſtant ſickneſs at ſtomach, very little food of any kind could be got down. In a few days after, ſome of the nails and a little of the hair of a full grown fœtus, (as was judged from the ſize of the nails) were diſcharged from the vagina, and theſe were followed by ſome ſmall finger bones. The patient continued much in the ſame ſtate till about the middle of June, the diſcharge becoming, if poſſible, every day more offenſive. About this time, the appearance of this was changed ; it ſeemed now to conſiſt, at firſt, in part, and afterwards altogether, of a moſt intolerably ſtinking oil ; the quantity, however, diminiſhed daily, and by the end of the third week in June, it had nearly ceaſed to flow ; and the patient, in the mean time, began to recover appetite, and to feel a return of ſtrength.

By the 1ſt of July ſhe ſeemed much recovered in her looks ; the bloated face, and oedematous affection of the lower extremities, had intirely diſappeared ; the ſize of her belly was much leſſened ; the fetid diſcharge had intirely ceaſed, her appetite was good, and ſhe was able to ſit up a great part of the day. In ſhort, the poor woman ſeemed now to be in a ſtate of convaleſcence, recruiting faſt, both in ſpirits and ſtrength, when unfortunately ſhe was perſuaded by ſome fooliſh adviſer, that a good jumbling in a coach would bring on her long expected labour, and rid her at once of her remaining incumbrance; a coach was accordingly procured, and although the motion of it gave her excruciating pain, particularly about the navel, at which part ſhe had for ſome days felt a pricking pain upon bending her body forward, yet fully perſuaded, that it was from the ſhaking that ſhe was to expect a ſalutary effect, ſhe bore it with fortitude. The pain continued increaſing after her return home, with great ſoreneſs over the whole abdomen, and ſhe expired early in the morning on the 7th of July, two days after the fatal ride.

Leave being obtained to examine the body, the diſſection was accurately performed in the preſence of Mr. Curtis and myſelf, by Mr. Whately, from whoſe notes the following account was taken. When the crucial inciſion was made through the integuments of the abdomen, the lower flaps could not be turned down, on account of the adheſion of the peritoneal lining with the contiguous parts. But upon carefully diſſecting them away, there was immediately diſcovered nearly the whole of the bones of a fœtus, which appeared to be of the ordinary ſize of a child born at the full time, or perhaps ſomewhat ſmaller. Theſe bones bones were entirely deprived of all their coverings, were feparated from each other, and compleatly packed in a round form, about the fize of a man's fift, being glued together by a black fubftance not unlike pitch, fuppofed to be fome remains of the putrid foft parts. The bones of the fcull were not only feparated, but their convex fides turned downwards, fo as to form a fort of cup or fmall bafon, which received the other bones, and refting upon the brim of the pelvis, prevented them from falling lower. This mafs of bones was furrounded by a membranous fac, of a black colour, which was perfectly fmooth on its internal furface, and adhered externally to all the contiguous parts. By thefe means a complete cavity was formed to contain the bones, which had no communication with that of the abdomen. The anterior part of this cavity was bounded by the pofterior furface of the bladder, and the peritoneal lining of the parietes of the abdomen as high as the navel; the fuperior and lateral portions, by the contiguous parts of the inteftines and omentum, cemented together by preternatural adhefions; the pofterior portion, by the anterior furface of the womb, and by the great arch of the colon, dragged down out of its natural fituation, and firmly attached tached, by its inferior border, to the fundus uteri; by which means all communication was excluded between the cavity containing the bones, and that part of the pelvis containing the rectum, which of courfe paffed in its natural fituation, without any preternatural adhefion, or other morbid appearance, from which the pofterior furface of the uterus was likewife perfectly free. All thefe parts every where adhered to, and were lined by the above mentioned membrane. Perhaps it may affift in giving a clearer idea of this cavity, to compare it to a blown bladder—that of a fheep, for inftance, thruft into the pelvis with the fmaller end downwards, between the urinary bladder and the womb, and glued on the outfide to all the parts that came in contact with it: the mafs of bones contained within the bladder, being fupported in the upper part of it, by refting upon the brim of the pelvis, as above defcribed. By this fituation of the bones, there was neceffarily a void fpace below them, no part of any of the vifcera lying clofe to the lower part of the mafs. In the anterior part of the cervix uteri, next the bladder, was a rent through its fubftance, about three quarters of an inch in length, the fides of which were nearly contiguous contiguous, but ulcerated, and not difspofed to heal. Several fmall bones of the fingers and toes had fallen down from the mafs above, into the moft depending part of the cavity, and were found lying about this opening. The uterus, very little larger than it is ufually found in its unimpregnated ftate, was in its natural fituation in the pelvis; and the fallopian tubes and ovaries were without any morbid appearance. The vagina was perfectly entire, fo that there was no paffage for the diffolved putrid matter of the fœtus, and the fmall bones that were difcharged, but through this rent in the uterus, and thence through the mouth of the womb into the vagina. Thofe portions of the inteftines that adhered to the fac were rather conftricted in their canal, and had evident figns of inflammation on their furface. No mark of difeafe was obferved in the other vifcera, except that the liver was larger, firmer, and of a much yellower colour than is ufual; and the gall-bladder was much diftended with bile, but without obftruction in the ducts. Juft below the navel were two fmall perforations of the membranous fac, through which the rough ends of two of the bones of the extremities tremities could be ſeen before the ſac was laid open.

This caſe may, perhaps, be thought worthy to be recorded as a remarkable proof of the powers of nature, in inſtituting new proceſſes in the animal economy, not only to preſerve life, but to get rid of whatever may impede the due exerciſe of the functions, and alſo as an inſtance of a ſurviving, for a conſiderable time, an accident that is uſually mortal. A rupture of the womb, is known to be, for the moſt part, ſpeedily fatal; yet there have not been wanting inſtances of a fortunate recovery from this accident, where a timely delivery has been effected by art, as in a remarkable caſe publiſhed by my friend and late colleague, Dr. Douglas; and this caſe ſhows the poſſibility of an eſcape, even where the child has been ſuffered to remain in the cavity of the abdomen; for although the event was at laſt unfortunate, yet there is reaſon to believe, that, under more favourable circumſtances, perhaps, even if all violent motion had been avoided, it might have terminated happily. Conſiderable progreſs was made towards a cure, and the remaining ſteps neceſſary to complete complete the recovery are explained by the diffection. All the foft parts of the fœtus, and even the cartilages had been diffolved by putrefaction ; to which procefs the fat feemed to be the leaft difpofed, being difcharged in the form of oil, after all the flefh was diffolved. The putrefcent matter being thus got rid of, and the patient's ftrength, in confequence, daily recruiting, the bones only remained to be expelled: A procefs for this purpofe feemed to be already commenced; the rough extremities of two of the bones had begun to irritate the integuments of the abdomen, at the navel, and would probably have made an opening there, at which the principal part of the bones might have been evacuated. The remainder which had already, or might afterwards have fallen to the bottom of the cavity, would either have made their way through the rupture of the uterus, and have been difcharged as the others were, or, by their conftant irritation, might have occafioned a more immediate opening into the vagina, or the rectum. The membranous fac lining the cavity which contained the bones, would have protected the inteftines and other vifcera, from the immediate contact of the external air. Was this fac

ſac entirely adventitious, and formed by a proceſs of nature? or could it conſiſt of the membranes naturally inveſting the child, retaining, by means of adheſion to the contiguous parts, enough of the principle of life to enable them to withſtand the putrefactive proceſs. The uniform black appearance of it may ſeem to countenance the laſt opinion; and although it ſeems moſt probable, that the membranes would give way at the part where they met with the leaſt ſupport, that is at the rupture, yet when we conſider the proportion that three quarters of an inch bear to the whole length of the uterus in its contracted ſtate, it is not improbable that the rent might at firſt exceed ſix inches; and through ſo large an opening, it is eaſy to conceive that the membranes might remain entire before the child, and tear off at the edge of the placenta, as we ſee often happens in quick labours; in which caſe the child might remain partly inveſted with its membranes. It ſeems even poſſible, that the whole ovum may have eſcaped entire into the abdomen.

A queſtion may ariſe, at what time the rupture of the uterus took place; but if the midwife's account is to be credited, the child muſt have eſcaped from the womb after ſhe ſaw her, and the ſize of the bones ſeems to confirm her account; otherwiſe the hemorrhage that took place immediately after the violent exerciſe, and did not return afterwards, would lead to a ſuſpicion that the uterus was ruptured at that time; and the adheſion of the lower border of the great arch of the colon to the fundus uteri, ſeems to ſhow that ſome inflammation, at leaſt, was brought on the womb at that time.

A knowledge of this caſe has led me to ſuſpect that many of the ſuppoſed inſtances of extra-uterine geſtation, have, in reality, been caſes of ruptured uterus; and I have little doubt of referring hither, a ſuppoſed ventral geſtation, of which an account has been lately publiſhed. For had the rent in my patient's womb healed by the firſt intention, (and by the cloſe contact of its ſides, it appears that this might have very readily happened;) all acceſs of the external air being prevented, the child would probably have remained long in the cavity of the abdomen, without undergoing much putrefaction: And, upon examination after death, who would then have ſuſpected that this child had ever been within the cavity of the womb.

XVI. *Caſe*

